# Youth Psychopathology in Daily Life: Systematically Reviewed Characteristics and Potentials of Ecological Momentary Assessment Applications

**DOI:** 10.1007/s10578-021-01177-8

**Published:** 2021-06-01

**Authors:** Marjolein R. Thunnissen, Marije aan het Rot, Barbara J. van den Hoofdakker, Maaike H. Nauta

**Affiliations:** 1 Department of Clinical Psychology and Experimental Psychopathology, University of Groningen, Grote Kruisstraat 2/1, 9712 TS Groningen, The Netherlands; 2 University Center for Child and Adolescent Psychiatry, Accare, Groningen, The Netherlands; 3 Department of Child and Adolescent Psychiatry, University Medical Center Groningen, University of Groningen, Groningen, The Netherlands

**Keywords:** Ecological momentary assessment, Youth, Psychopathology, Internalizing disorders, Externalizing disorders

## Abstract

Traditionally, symptoms of youth psychopathology are assessed with questionnaires, clinical interviews, or laboratory observations. Ecological Momentary Assessment (EMA) could be a particularly valuable additional methodology, since EMA enables examining the daily lives of youths near real-time, considering fluctuations and specific contexts of symptoms. This systematic review aimed to review the characteristics of current EMA applications and to provide a synthesis of their potential in studying youth psychopathology. Following a systematic search in PsycInfo and Medline, we identified 50 studies in clinical samples. Most studies used EMA to examine fluctuations in symptoms, affect, and behavior, and the relation with contextual factors. EMA was also used to investigate interactions between parents and their children over time, and to monitor and predict treatment response. EMA appeared feasible in youth and could provide valuable insights that contribute to understanding youth psychopathology. Benefits, gaps, and suggestions for future research and clinical practice are discussed.

**Supplementary Information:**

The online version contains supplementary material available at 10.1007/s10578-021-01177-8.

## Introduction

Youth psychopathology is common [1–3]. For example, Kessler et al. [2] found 12-month prevalence estimates of psychopathology as high as 40% in adolescents. Traditionally, symptoms of psychopathology are assessed retrospectively, typically covering intervals of between one-two weeks (in the case of questionnaires) to up to twelve months (in the case of clinical interviews). Although real-time laboratory observations may provide a reasonable alternative to retrospective studies, these approaches also have disadvantages. Specifically, they fail to provide insights into important aspects of real-life daily functioning, the natural context in which symptoms occur, and variability of symptoms over time.

Methods that enable examining the daily lives of youth prospectively, such as Ecological Momentary Assessment (EMA) and related techniques, could be of additional value in understanding youth psychopathology. EMA is a method characterized by assessing individuals at multiple time points in their natural environments, often to examine their current or recent moods or behaviors [4, 5]. The development of psychopathology in youth is influenced by their families and peers, early childhood stressors, disease, and other environmental factors. More specifically, children and adolescents develop in the context of different environments including their home, school and peers, and symptoms may both be influenced by various environmental factors and influence them. As it is considered critical to consider these specific contextual influences when studying youth [6], EMA may be of particular importance for research on psychopathology in children and adolescents.

Furthermore, the clinical presentation of symptoms may vary with age, and symptoms may occur mainly in the context of specific situations, events, or relationships [7]. Thus, sampling environmental changes over time may also be very helpful for understanding psychopathology and examining treatment outcomes. Similarly, being able to capture variability in moods and behaviors and the temporal relations between these types of variables is of great significance. What is considered typical for a child or adolescent depends on what would be expected from the cognitive and emotion regulation abilities of youth in different developmental periods. These abilities are expected to change over time [6]. This is reflected in the criteria for diagnosing psychopathology in youth. For example, specific behaviors are formulated for children in the context of specific phobias, noting that a child may express fear or anxiety by crying, tantrums, freezing, or clinging [8]. EMA is well suited to assess variability and changes over time in relevant variables, as well as temporal relations between variables.

EMA may also be of additional value in understanding youth psychopathology, considering that children may not always adequately report on their moods or behaviors retrospectively compared to adults [9], and may be more apt to report on the here-and-now. In general, conventional retrospective assessments risk obtaining biased responses caused by, for example, the personal heuristics effect, the recency effect, the salience or novelty effect, or the mood-congruent memory effect [10] (for an example, see [11]). In addition, the increase in employing mobile-device-based EMA for reports over shorter recall periods may be well suited for youths; they appear to be particularly skilled in using mobile technology and may benefit from device reminders. However, differences may exist in the suitability of EMA techniques between adolescents and school-age children.

Depending on the specific psychopathology and research question, EMA can be contingent upon the occurrence of specific times, (pseudo-)random signals, or certain events [4, 5]. With time-contingent variants, assessments occur at regular pre-specified time points (triggered by a device if mobile technology is used). With signal-contingent variants, participants receive signals at more or less random time points and then complete assessments. Both variants are used for assessing variability of mood and behavior [12] as well as for examining temporal relations between variables. With event-contingent EMA, participants are instructed to complete the assessments after certain well-defined events such as social interactions, binge eating episodes, or substance use. Event-contingent EMA is best used when psychopathology symptoms are tied to specific (interpersonal or other) contexts. Different designs can be combined within one study, depending on the specific research question [7, 12]. EMA measurement intervals can thus be adapted and EMA can be conducted using, for example, paper diaries, mobile phone calls or smartphone applications. Recent developments in and increased availability of mobile technology allow for even more possibilities in data collection.

In sum, EMA provides specific opportunities for studying psychopathology in youth, as it can be used to examine the context and fluctuations of symptoms in daily life, while minimizing bias and allowing adaptability to the specific topic of interest. EMA may therefore be a useful additional tool in analyzing the clinical presentation and course of youth psychopathology, interactions between symptoms and environmental factors, and treatment outcomes. Consequently, the aims of the present systematic review are to review the characteristics of current EMA applications and to provide a synthesis of their potential in studying youth psychopathology.

To the best of our knowledge, there have been no previous systematic reviews that covered the applications of EMA for the study of all youth psychopathology (including both internalizing and externalizing disorders). There have been systematic reviews on the use of EMA for the study of adults with psychopathology (e.g., [13]) or for the study of specific types of psychopathology, regardless of age (e.g., mood disorders [14] and attention-deficit/hyperactivity disorder (ADHD) [15]). In the present review, by considering the broad range of psychopathology in youth, researchers and clinicians can be informed of and inspired by the use of EMA in various fields. While a systematic review has been conducted on methodological approaches and implementation challenges in using mobile-technology-based EMA in youth [16], this review did not specifically focus on the potential of using EMA to study youth psychopathology. Our systematic review adds to that and aims (1) to review the characteristics of current EMA applications and (2) to provide a synthesis of their potential in studying youth psychopathology in terms of (a) feasibility and validity of EMA in youth with psychopathology, (b) studying the phenomenology of youth psychopathology and its correlates in daily life, including parent–child interactions, and (c) using EMA in evaluating treatment outcomes. Accordingly, the results section involves a review of the characteristics of how EMA has been used so far across disorders and how this use has shown potential in studying youth psychopathology rather than a review of the results of EMA of specific variables in youth with mental disorders.

## Method

We conducted a search in the PsycINFO and MEDLINE databases combining terms used to describe EMA methodology with a listing of mental disorders and terms reflecting youth: (“diary” OR “momentary assessment” OR “experience sampling” OR “event-contingent recording” OR “ambulatory assessment”) AND (“anxi*” OR “phobi*” OR “agoraphobi*” OR “panic” OR "posttraumatic stress" OR “PTSD” OR “OCD” OR “obsessive compulsive” OR “GAD” OR “depress*” OR “MDD” OR “affective disorder*” OR “mood disorder*” OR “bipolar” OR “ADHD*” OR “attention-deficit hyperactivity” OR “conduct disorder” OR “ODD” OR “oppositional defiant” OR “eating disorder*” OR “anore*” OR “bulimi*” OR “binge eating” OR “substance use” OR “substance abuse” OR "substance dependen*” OR “autis*” OR “asperger” OR “PDD-NOS” OR “pervasive developmental” OR “psychosis” OR “psychotic” OR “schizo*” OR “enuresis” OR “encopresis” OR “tic” OR “tourette” OR “somatization disorder*” OR “pain disorder*” OR “conversion disorder*” OR “hypochondria*” OR “body dysmorphic” OR “sleep disorder*”) AND (“child*” OR “adolescen*” OR “pediatric” OR “youth”).

We executed the search on 16 April 2020. We set limits for publication date (from 1994 to 2020), language (English), and publication type (dissertations excluded), in line with the following selection criteria. First, the study had to be empirical in nature. Second, participants in the study had to be younger than 18 years of age (on average). Third, we only included studies targeting a clinical sample, defined as having a diagnosis of an Axis I disorder in the Diagnostic and Statistical Manual of Mental Disorders, 4th edition (DSM-IV) or a comparable DSM-5 disorder [8, 17]. In line with previous EMA reviews, studies using older DSM editions were not included, also considering the paucity of EMA studies before the release of the DSM-IV in 1994 [12]. Fourth, studies had to use EMA or related intensive repeated methods, with more than one assessment per day and more than one day (24 h) of assessment. This was also in line with previous reviews. Finally, the EMA outcome variables had to be assessed in the context of studying youth symptomatology and include affective, behavioral, cognitive, interpersonal, or biological measures. Therefore, we excluded studies that only provided a methodological report, or, in case of parent-report, that focused on parental outcome variables alone. We also excluded studies that solely included retrospective sleep diaries, and studies that were restricted to assessing food intake, voiding or defecation if not used to examine psychopathology. Thus, for example, when food intake was assessed to study disorder-related behavior (e.g., binge eating), the study could be included.

The articles found by the search were first subjected to title-based selection. The second step was abstract-based selection of the selected titles and the final step was selection of the remaining studies based on the method section. All steps were taken twice, independently, by the first and the last author, or by the first author and a research assistant, using the aforementioned selection criteria. In the first two steps, all papers that were included by at least one of two reviewers were selected for the next step. For the final inclusion, disagreements between the two reviewers were resolved by reaching consensus during a discussion meeting. We also checked reference lists of included papers for other relevant sources, and followed the selection procedure as described above.

Given our first aim of reviewing the characteristics of EMA applications, we retrieved and reviewed data referring to the study sample (participants, age range, and DSM diagnosis of patient group, and, when applicable, participants and description of comparison groups) and the EMA procedures (format, contingency, completed by whom, observations per day, days, sum of observations, and outcome variables). Given our second aim of synthesizing the potential of applying EMA, we formed a narrative review of the data on EMA applications and relevant empirical data according to one of the three sub-topics. First, we summarized studies that specifically examined feasibility and validity, plus a synthesis on compliance with EMA measures. Second, we grouped studies that investigated the phenomenology of youth psychopathology. Third, we identified all treatment studies. Due to the large number of studies within the topic of phenomenology, this topic was organized per DSM section.

## Results

### Search Results

**Fig. 1:**
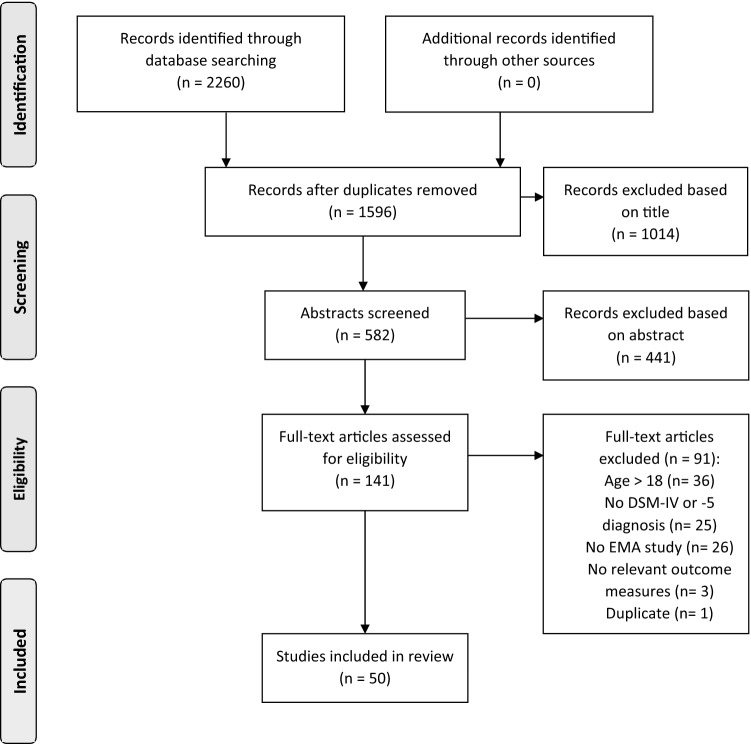
Flow diagram of the selection procedure using the Preferred Reporting Items for Systematic Reviews and Meta-Analyses (PRISMA) Guidelines

The initial search resulted in 1596 articles. Finally, 50 studies were included in our review (see PRISMA flowchart in Fig. 1 for an overview of the selection procedure).

### Characteristics of Current EMA Applications in Studying Youth Psychopathology

In Table 1, we describe the characteristics of the patient group and, if applicable, comparison groups, and details of the EMA procedures for all included studies. Of all studies, 15 studies examined ADHD, 5 autism spectrum disorders (ASD), 12 mood disorders (4 of which also examined anxiety disorders [18–21]), 16 anxiety disorders, 5 eating disorders, and 1 psychosis. The studies on ADHD considered 11 different samples of children and 1 sample of adolescents. The studies on ASD included 3 samples of children and adolescents with high-functioning autism spectrum disorder (HFASD). All studies on mood disorders involved children and adolescents with major depressive disorder (MDD); 1 study also included youth with bipolar disorder (BD). The studies on anxiety disorders mainly concerned children and adolescents with generalized anxiety disorder (GAD), separation anxiety, or social anxiety disorder (SAD). All 5 studies on eating disorders investigated adolescents with anorexia nervosa (AN), with (partial) overlap between study samples. The one study on psychosis considered adolescents with early-onset psychosis. Both school-age children (mostly between 7 and 12 years of age) and adolescents (mostly > 12 years) were studied in the context of the above-mentioned disorders, with the exception of eating disorders and psychosis (adolescents only). Sample sizes were variable, ranging from less than 20 (in case reports or pilot studies) to 133 youths with a mental disorder. Most studies (34) included a healthy or community-based comparison group; 3 studies also included a comparison group with other DSM diagnoses.

**Table 1:** Description of the studies included in the systematic review

Authors	Patient group			Comparison group(s)		EMA details							
	*N*	Age range	DSM diagnosis	*N*	Description	Format	Contingency	Completed by	Observations/day	Days	Observations	Compliance^1^	Outcome variable(s)
Attention-deficit/hyperactivity disorder													
Babinski and Welkie [27]	13	12–16	ADHD-only, ADHD-comorbid behavioral, mood or ANX disorder	–	–	Mobile phone app	Signal	Parent and child	2–4	7	18	91% (parent) and 84% (child)	Negative emotion
Factor et al. [33]	42	8–12	ADHD-only, ADHD-comorbid behavioral, mood or ANX disorder	22	Healthy controls	PDA	Time	Parent and child	3	28	84	76% (parent)	Affect
Fogleman et al. [42]	59	8–12	ADHD	45	Community sample	PDA	Time	Parent	3	28	84	83%	Affect
Leaberry et al. [34]	58	8–12	ADHD-only, ADHD-comorbid behavioral, mood or ANX disorder	–	–	PDA	Time	Parent	3	28	84	81%	Affect
Rosen and Factor [25]	27	8–11	ADHD (incl. comorbid behavioral, mood or ANX disorder)	–	–	PDA	Time	Parent and child	3	28	84	85% (parent) and 77% (child)	Affect
Rosen et al. [26]	11	8–11	ADHD (incl. comorbid behavioral, mood or ANX disorder)	–	–	PDA	Time	Parent and child	3	28	84	87% (parent) and 77% (child)	Affect
Rosen et al. [35]	56	8–12	ADHD (incl. comorbid behavioral, mood or ANX disorder)	46	Community sample	PDA	Time	Parent	3	28	84	82%	Affect
Slaughter et al. [36]	53	8–13	ADHD (incl. comorbid behavioral, mood or ANX disorder)	43	Community sample	PDA	Time	Parent	3	28	84	Unclear	Affect
Walerius et al. [37]	47	8–12	ADHD (incl. comorbid behavioral, mood or ANX disorder)	37	Community sample	PDA	Time	Parent	3	4–10	12–31	85%	Affect
Walerius et al. [38]	42	8–12	ADHD (incl. comorbid behavioral, mood or ANX disorder)	32	Com–munity sample	PDA	Time	Parent	3	28	84	89%	Affect, functional impairment
Whalen et al. [63]	51	8–12	ADHD	58	Healthy controls	PDA	Time	Mother	2	7	14	99%	Mood, behavior
Whalen et al. [40]	51	8–12	ADHD	58	Healthy controls	PDA	Signal	Mother and Child	Every 30 min in monitoring intervals	7	Unclear	93–94% (mother) and 91–94% (child)	Anger, stress, good mood
Whalen et al. [39]	27	7–12	ADHD	25	Healthy controls	PDA	Signal	Mother and Child	Every 30 min in monitoring intervals	7	Unclear	91–92% (mother) and 89–90% (child)	Behaviors, context, mood, interaction quality
Whalen et al. [32]	27	7–12	ADHD	25	Healthy controls	PDA	Signal + time	Mother and Child	Every 30 min in monitoring intervals + mothers 2/day	7	Unclear	91–92% (mother) and 89–90% (child)	Behaviors, context, mood, interaction quality, parenting
Whalen et al. [41]	51	8–12	ADHD	58	Healthy controls	PDA	Signal	Mother and Child	Every 30 min in monitoring intervals	7	Unclear	93–94% (mother) and 91–94% (child)	Behaviors, mood, context
Autism spectrum disorders													
Chen et al. [23]	6	8–12	HFASD	–	–	iPod Touch app	Signal	Child	7	7	49	57%	Affect, social context, quality of experiences
Cordier et al. [44]	6	8–12	HFASD	–	–	iPod Touch app	Signal	Child	7	7	49	57%	Affect, social context, quality of experiences
Khor et al. [22]	31	12–18	HFASD	–	–	Mobile phone app	Signal	Child	4 (1 on the final day)	14	53	62%	Stress
Khor et al. [43]	31	12–18	HFASD	–	–	Mobile phone app	Signal	Child	4	14	53	62%	Stress
Kovac et al. [24]	19	9–19	HFASD	20	Healthy controls	Smartphone or computer web survey	Signal	Child	1–2	4	6	85–93%	Affect, behavioral and social context
Mood disorders and anxiety disorders													
Allen et al. [29]	96	4–15	Separation anxiety (incl. comorbid ANX, MDD, externalizing disorder)	92	Healthy controls and other ANX	Diary	Event	Mother	1 or more	8	8 or more	90% (overview) and 68% (situation sheets)	Separations, anxiety, thoughts, behaviors, parental reactions
Allen et al. [30]	58	7–14	Separation anxiety (incl. comorbid ANX, MDD, externalizing disorder)	67	Healthy controls and other ANX	Diary	Event	Child	1 or more	8	8 or more	89% (overview) and 71% (situation sheets)	Separations, anxiety, thoughts, behaviors, parental reactions
Allen et al. [52]	106	9–14	ANX (incl. comorbid ANX, externalizing disorder)	–	–	Phone call	Signal	Child	2–4	5	14	51% of calls included	Perceived control, emotional reactivity, emotion regulation
Axelson et al. [28]	16	10–17	MDD, BD, lifetime GAD/MDD	5	Healthy controls	Phone call	Signal	Child	2–4	20	60	90%	Affect, behavior, motivation, social context
Beidel et al. [50]	50	7–13	Social anxiety (incl. comorbid ANX, MDD, externalizing disorder)	22	Healthy controls	Diary	Event	Child	1 or more	14	14 or more	86%	Socially distressing events, coping behavior
Butterfield et al. [57]	87	9–14	ANX (incl. comorbid ANX, MDD, externalizing disorder)	33	Healthy controls	Phone call	Signal	Child	2–4	5	14	Unclear	Distressing events, coping behavior
Cousins et al. [19]	65	8–16	MDD, ANX, comorbid MDD and ANX	29	Healthy controls	Phone call	Signal	Child	2–4	8	24	92%	Affect, sleep
Doane et al. [21]	114	16–18	Past or recent MDD or ANX, comorbid MDD and ANX	186	Healthy controls	Digital watch + diary, vial	Signal + time	Child	6	3	18	Unclear	Affect, cortisol (saliva sample into vial)
Forbes et al. [49]	15	8–17	MDD (incl. comorbid ANX)	28	Healthy controls	Phone call	Signal	Child	2–4	4	12	95%	Affect
Forbes et al. [20]	66	8–16	MDD, ANX, comorbid MDD and ANX	–	–	Phone call	Signal	Child	2–4	20	60	89%	Affect, social context
Mor et al. [18]	47	16.9 (*M*)	MDD, ANX, comorbid MDD and ANX	231	Healthy and other DSM controls	Digital watch and diary	Signal + time	Child	6	3	18	91%	Affect, stress, self–focus
Morgan et al. [54]	130	9–14	Social anxiety, ANX (incl. comorbid ANX, MDD, externalizing disorder)	46	Healthy controls	Phone call	Signal	Child	2–4	25	70	82% (social anxiety) and 77% (ANX) –78%	Affect, social context
Price et al. [56]	78	9–14	ANX (incl. comorbid ANX, MDD, externalizing disorder)	20	Healthy controls	Phone call	Signal	Child	2–4	5	14	56% of calls included	Affect, emotion regulation
Primack et al. [45]	46	7–17	MDD (incl. comorbid ANX)	60	Healthy controls	Phone call	Signal	Child	2–4	20	60	89%	Media exposure
Silk et al. [48]	20	8–17	MDD (incl. comorbid ANX)	22	Healthy controls	Phone call	Signal	Child	2–4	4	12	Unclear	Affect
Silk et al. [64]	47	7–17	MDD (incl. comorbid ANX)	32	Healthy controls	Phone call	Signal	Child	2–4	20	60	92%	Affect, behavior, social context
Silk et al. [66]	133	9–14	ANX (incl. comorbid ANX, MDD, externalizing disorder)	–	–	Phone call	Signal	Child	2–4	25	70	89%	Affect
Smith et al. [55]	37	8–18	ANX (incl. comorbid ANX, MDD, externalizing disorder)	20	Healthy controls	Mobile phone app	Time	Child	3	7	21	76%	Affect, interactions with peers
Stone et al. [53]	117	9–14	ANX (incl. comorbid ANX, externalizing disorder)	–	–	Phone call	Signal	Child	2–4	5	14	29% of calls included	Affect, social context, emotion regulation
Tan et al. [51]	65	9–13	ANX (incl. comorbid ANX, externalizing disorder)	65	Healthy controls	Phone call	Signal	Child	2–4	5	14	93%–91%	Affect, emotion regulation
Teixeira and Freire [65]	1	14	MDD	–	–	Electronic device + diary	Signal	Child	8	21	168	65%	Mood, self–satisfaction, context
Wallace et al. [67]	114	9–14	ANX (incl. comorbid ANX, MDD, externalizing disorder)	–	–	Phone call	Signal	Child	2–4	5	14	92%	Affect, social context, events, sleep
Waller et al. [46]	29	11–17	MDD (incl. comorbid ANX, externalizing disorder)	31	Healthy controls	Phone call	Signal	Child	3	15	42	83%	Social context, problem talk
Whalen et al. [47]	30	7–17	MDD (incl. comorbid ANX, externalizing disorder)	23	Healthy controls	Phone call	Signal	Child	2–4	20	60	Unclear	Affect, caffeine consumption, sleep
Eating disorders													
Fürtjes et al. [62]	33	12–19	Anorexia nervosa (incl. comorbid MDD, ANX)	–	–	Mobile phone app	Signal	Child	6	28	168	82%	Rumination, affect
Kolar et al. [58]	20	12–19	Anorexia nervosa (incl. comorbid MDD, ANX, BD)	20	Healthy controls	Mobile phone app	Signal	Child	Every full hour	2	25–26 (mean)	79%–82%	Aversive tension
Kolar et al. [59]	20	12–19	Anorexia nervosa (incl. comorbid MDD, ANX, BD)	20	Healthy controls	Mobile phone app	Signal	Child	Every full hour	2	25–26 (mean)	79%–82%	Emotion identification, aversive tension
Seidel et al. [60]	37	12–28	Anorexia nervosa (incl. comorbid MDD, ANX)	33	Healthy controls	Mobile phone app	Signal	Child	6	14	84	84%–76%	Rumination, affect
Seidel et al. [61]	35	12–29	Anorexia nervosa (incl. comorbid MDD, ANX)	35	Healthy controls	Mobile phone app	Signal	Child	6	14	84	84%–76%	Rumination, affect
Psychosis													
Smelror et al. [31]	3	17.7 (*M*)	Early onset psychosis	–	–	iPod touch app	Signal + event	Child	5	7	35	74%	Auditory verbal hallucinations

*ADHD* attention-deficit/hyperactivity disorder, *ANX* anxiety disorder, *PDA* personal digital assistant, *HFASD* high-functioning autism spectrum disorder, *MDD* major depressive disorder, *BD* bipolar disorder, *GAD* generalized anxiety disorder, *app* application

^1^If two unspecified percentages are shown, distinct percentages were provided for the patient group and the comparison group, respectively

Youth completed EMA in 34 studies, their parents in 8 studies, and both in another 8 studies. EMA completed by both youth and their parents was used in the context of studying ADHD, and EMA completed only by parents about their child in ADHD and once in anxiety disorders. Various formats were used to obtain data: phone calls (17 studies); applications installed on a personal digital assistant (PDA) (14 studies), smartphone (9 studies), or iPod touch (3 studies); diary booklets with (3 studies) or without (3 studies) digital wristwatch or device; and online surveys on a smartphone or computer (1 study). One study also used vials to collect saliva samples for cortisol assessment. The use of phone calls and diary booklets occurred in the context of studying mood and anxiety disorders. Mobile technology applications or web surveys were also used once in this context, and were the only format used to study other disorders. Signal-contingent assessment was used most often (36 studies), followed by time-contingent assessment (14 studies) and event-contingent assessment (4 studies; with events being separations and socially distressing events in anxiety disorders, and auditory verbal hallucinations in psychosis). The 3 studies that were both time- and signal-based, and the 1 study that was both signal- and event-based were counted twice. The number of assessments ranged from 2 per day to every full hour (except sleeping hours) for periods of 2 to 28 days, resulting in 6 to 168 assessments across studies.

### Potential of EMA in Studying Youth Psychopathology

#### Feasibility and Validity

Data on compliance (i.e., data relating to the percentage of completed assessments) was available in 42 of the included studies and is included in Table 1. In these studies, compliance was generally satisfactory, both with parent and child report (ranging between 57 and 99%, and above 70% in 36 studies). There were some exceptions (e.g., a study with signal-contingent EMA in children with ASD reported 57%). Compliance was unclear in 5 studies. In the remaining 3 studies, only the percentage of included calls was described.

In addition to providing data on compliance, part of the included studies specifically examined the feasibility and validity of the EMA applications that were used (i.e., in ASD [22–24], ADHD [25–27], mood disorders [28], anxiety disorders [29, 30], and psychosis [31]). Based on these studies, we provide an initial review of the feasibility and validity of the EMA measures.

Overall, the studies provided support for the feasibility of the different applications, albeit with some relevant observations and concerns. The generally satisfactory results with regards to EMA compliance were accompanied by positive participant feedback. Youth with mood disorders reported positive feedback and willingness to complete signal-contingent EMA for 20 days [28]. Similarly, two studies that applied signal-contingent EMA in youth with HFASD implied overall satisfaction with questionnaire content, response options, and length, albeit with some exceptions [22, 23].

Possible explanations for non-compliance were also explored. In youths with HFASD, reasons for non-completion included being immerged in other activities and forgetting the device [23]. Furthermore, in the study on EMA of auditory verbal hallucinations (AVH) in adolescents with early-onset psychosis [31], adolescents indicated several concerns in an interview after the EMA, such as increased awareness of AVH and concerns about privacy. It was also noted that two of the three adolescents completed the sampling protocol, with the reason for non-completion being partly related to having to carry around the extra device for the sampling. The modest compliance rates of the mother and child event-based diaries on separations in the two studies in anxiety disorders were discussed in relation to possible forgetfulness, lack of motivation, or an inaccurate reflection of the mothers’ and children’s experiences in the preset diary options [29, 30]. The authors suggested that reminder beeps may improve compliance with this application.

EMA generally appeared to provide valid measurements of the variables of interest. In youth with mood disorders, temporal patterns and variability in the EMA data related to their symptoms could be demonstrated for individual cases [28]. For instance, EMA revealed large within-person variation in emotions over an 8-week course in one individual with BD [28]. Further, measures of child emotional variability derived from parent-reported signal- and time-contingent EMA data were found to be significantly associated with conventional measures of emotional and behavioral difficulties in children with ADHD, even after controlling for retrospective reports of emotion dysregulation [25]. Furthermore, EMA reports of negative emotion of adolescent girls with ADHD and their mothers were generally similar and showed significant moderate correlations [27]. Finally, in a study that used signal-contingent EMA in youths with HFASD, moderate to poor correspondence was reported between EMA and retrospective data, with EMA data revealing information about experienced stressors that was not captured by the retrospective questionnaires [22].

However, some other observations on child-reported EMA draw a somewhat different picture. For instance, child self-reports of affective variability in children with ADHD were not significantly related to their emotional and behavioral difficulties as measured using conventional measures [25]. Moreover, children’s affect ratings were more likely to be missing when the child was more distressed according to the parent reports, and generally differed considerably from parent ratings [25, 26]. Additionally, Rosen et al. [26] also found that children provided dichotomous and positively skewed answers.

#### Phenomenology and Its Correlates

Most of the EMA studies included in our review focused on examining the phenomenology of psychopathology and its correlates in daily life, and employed various EMA applications in youths with ADHD [32–42], ASD [24, 43, 44], mood disorders [18, 19, 21, 45–49], anxiety disorders [50–57], and eating disorders [58–62]. We discuss the multiple potential uses in these studies per DSM section.

##### Attention-Deficit/Hyperactivity Disorder (ADHD)

EMA was used to provide information about the phenomenology of ADHD by assessing fluctuations, temporal patterns, and contexts of affect and symptomatic behavior. In one of these studies, child mood was assessed at different times of the day using signal- and time-based mother and child reports, and these momentary reports were compared for children with and without ADHD [32]. In addition, comparisons were made between the temporal patterns during the week and in the weekends. In other studies, EMA was applied to assess emotional variability and to relate EMA-derived measures of child emotional variability to diagnostic status (i.e., the presence of ADHD and comorbid DSM-diagnoses) [33, 34], to conventional measures of emotional and behavioral difficulties [35, 36], to parenting hassles at one-week follow-up [37], and to daily functional impairment [38]. For example, it could be demonstrated that daily negative emotional lability predicted reactive aggression at both baseline and 6-month follow-up [36].

Moreover, EMA was successfully used to examine parent–child interactions in daily life and to investigate temporal links between parent and child moods and behaviors. One study, using signal-based EMA in mothers of children with and without ADHD, compared preparatory and transitional activities with other activities and examined shifts from positive to neutral or problematic interactions when engaging in these “getting-ready activities” [39]. In a similar study that focused on examining momentary anger reports of mothers and their children with and without ADHD [40], temporal links were investigated in terms of the temporal proximity of mothers’ reports of their own anger to anger reports of their children, and the speed of recovery of anger reported by mothers following child-reported anger. Thereby, the authors were able to show a slower recovery of anger in mothers of children with ADHD. In another study, EMA was used to examine fluctuations over time in levels of parenting distress and child- and mother-reported child behavioral problems [41]. This allowed for comparing the momentary synchrony of parental stress and child symptomatic behaviors between parents and their children with and without in the ADHD. Lastly, one study related daily reports of negative affect to retrospective reports of peer victimization in both children with and without ADHD [42].

##### Autism Spectrum Disorders (ASD)

EMA was applied to provide information about the phenomenology of ASD by using signal-based EMA to assess affect, coping, and related social and behavioral contexts [24, 43, 44]. One study assessed the relation between positive affect and real-world behavioral contexts [24], and another study assessed the quality of everyday social experiences from the perspective of youth with HFASD [44]. In a third study, momentary coping strategies were assessed in relation to behavior and emotional problems [43]. For example, momentary disengagement coping in particular (i.e. avoidance, denial, wishful thinking) was prospectively associated with more behavioral and emotional problems two weeks later [43].

##### Mood Disorders

EMA was used to study the phenomenology of mood disorders by applying signal- and time-based EMA of the real-world context of problematic thoughts, behaviors and affect, and the differing social lives of youth. In one of these studies, Mor et al. [18] used EMA to examine within-person variability of self-focus, negative affect, and stress in youths with MDD and healthy controls. In another study, EMA by frequent phone calls was used to assess the number of hours spent on media, including internet, television, and video games, and to examine the association between MDD and media exposure in daily life [45]. Waller et al. [46] described how they applied EMA to gather in-depth data on the social context of behavior in youth: rates of co-rumination and co-problem solving during social interactions were assessed in youth with MDD and healthy controls. Furthermore, using EMA, caffeine consumption and its links to sleep and affect were examined in youth with MDD [47].

EMA was also successfully used in combination with real-life physiological measures; for instance, EMA-measured affect was linked to actigraph-measured sleep [19]. The authors used this data to examine the bidirectional relation between daily affect and sleep or time in bed. Doane et al. [21] examined the association between cortisol diurnal activity and psychopathology: they related past MDD, recent comorbid MDD, and anxiety to cortisol slopes assessed in everyday life. Also, data on daily negative emotions was related to diurnal cortisol rhythms.

In other studies, physiological variables measured by laboratory paradigms were related to real-world affect. In one study, pupil dilation to negative words in a lab was related to mood in daily life [48]. Another study investigated activation in caudate regions of the brain, as measured by a laboratory-based reward paradigm, in youth with MDD and a control group, and related this specific brain activation to EMA-data on positive affect in daily life [49].

##### Anxiety Disorders

Both event- and signal-based applications of EMA were used to study the phenomenology of anxiety disorders by assessing real-life distressing events and associated coping and emotion regulation strategies. In a study on SAD, Beidel et al. [50] used child event-based diaries to assess the range of social situations that were reported as causing distress, as well as related coping behaviors. Thereby, the authors were able to demonstrate that in approximately 35% of distressing events, children had a maladaptive response of avoidance such as pretending not to hear the person talking to them. Tan et al. [51] used signal-based EMA to compare the emotional reactivity and emotion regulation strategies of children with and without an anxiety disorder. They examined the frequency and range of emotion regulation strategies as well as the effectiveness of the strategies in lowering negative emotion in daily life.

Signal- and time-based EMA was also used to study interactions of youth with and without an anxiety disorder with their parents and peers. In one of these studies, Allen et al. [52] examined the association between parental autonomy granting, child perceived control, and everyday emotional reactivity and regulation in anxious youth. The effectiveness of daily emotion regulation strategies was investigated in another study, while taking social context (non-social, parents, or peers) into account [53]. Morgan et al. [54] examined the relations between positive affect, social context, and anxiety type. Thereby, they were able to demonstrate differences in peak positive affect between youth with SAD and healthy youth specifically during interactions with less close peers. Finally, ratings of peer interactions were related to both concurrent affect and affect at later time points [55].

Considering the relation between laboratory and real-world data, Price et al. [56] examined individual differences in attentional vigilance in the lab, related neural substrates, and real-world avoidance as an emotion regulation strategy after a negative event in youth with an anxiety disorder. Similarly, Butterfield et al. [57] related assessments of parental coping socialization to youth neural threat response and EMA data on disengaged coping.

##### Eating Disorders

Signal-contingent EMA was used to examine the phenomenology of AN by assessing negative affect and disorder-related rumination in daily life [60–62]. Seidel et al. [60] studied the associations between negative affect and tension and concurrent weight- and food-related rumination, and examined differences in the strength of this association between adolescents with and without AN. Furtjes et al. [62] repeated the same EMA protocol after weight restoration in the adolescents with AN, and investigated disorder-related rumination in relation to changes in biological markers of undernutrition. Furthermore, Seidel et al. [61] linked neural substrates of emotion (over)regulation to daily rumination and negative affect. In two other studies, signal-contingent EMA applications with shorter but more intensive assessment schedules were successfully used to assess momentary aversive tension [58] and emotion identification [59].

#### Treatment

A limited number of the included studies used EMA when evaluating treatment outcomes, and employed EMA applications in youths with ADHD [63], mood disorders [20, 64, 65], and anxiety disorders [66, 67]. We discuss the potential uses covered by these studies below.

First, one study used time-contingent EMA to study the effects of medication in ADHD [63]. They specifically applied EMA to investigate potential differences in functioning between day times during treatment. By conducting real-time assessments in the mornings and evenings, the authors were able to examine the effects of taking atomoxetine and long-acting stimulants on mood and behavior.

Second, one study examined momentary intensity and lability of negative affect, time spent alone, and the positive to negative affect ratio over the course of treatment for MDD [64]. This was done by applying repeated 5-day sampling blocks of signal-contingent EMA. Thereby, differences over the course of treatment between MDD groups and a control group could be examined. Similarly, a treatment study in youths with anxiety disorders applied EMA to investigate the differential impact of cognitive behavioral therapy (CBT) versus child-centered therapy on daily negative emotions [66]; the authors were able to show differences between the groups in peak negative emotions related to recent negative events in the second half of treatment.

A third application involved intensive signal-contingent EMA for closely monitoring treatment effects on an individual level. Teixeira and Freire [65] used this application in an adolescent girl with MDD in order to monitor her daily mood, mood stability, and diversity of activities closely. EMA was conducted at three different time points during treatment.

The fourth and final application was found in two studies that used signal-contingent EMA to identify potential treatment predictors or moderators. One study used EMA-measured positive and negative affect and social context at baseline to predict the treatment response of youth with affective disorders [20]. The authors were able to predict treatment response by these EMA-derived measures, over and above traditional symptom questionnaires. Another study similarly explored EMA-derived predictors as well as moderators of treatment outcome in youths with anxiety disorders [67].

## Discussion

The purpose of this systematic review was to review the characteristics of current EMA applications and to synthesize their potential in studying youth psychopathology. The reviewed studies considered youth with ADHD, ASD, mood and anxiety disorders, eating disorders, and psychosis, and applied a variety of EMA techniques. In the following text, we discuss the insights that our review provided in the light of the presumed benefits of EMA in studying youth psychopathology. We also discuss gaps, future directions for research and clinical practice and strengths and limitations of our review.

### Characteristics of Current EMA Applications for Studying Youth Psychopathology

The diverse possibilities of using EMA are underlined by the various EMA applications that were found for studying youth psychopathology, capturing a broad age range, several types of psychopathology, and different EMA procedures with regards to format, contingency, intensity and duration. The results of our review pointed out potential gaps regarding, for example, the use of event-based EMA or parent-report EMA across the types of psychopathology. Our review also indicated gaps with regards to the psychopathology types and age groups that were studied using EMA measures. For example, even though we included the broad spectrum of ASD and eating disorders in the search terms, only studies that examined youth with HFASD and adolescents with AN were found. Also, there was but a single pilot in early-onset psychosis. In comparison, psychotic disorder has frequently been studied using EMA in adults (e.g., [68]).

### Feasibility and Validity of EMA in Studying Youth Psychopathology

Considering our findings regarding compliance, EMA methods seem generally feasible in various populations of youth with psychopathology. In addition, EMA appeared to provide measures with adequate construct validity, as indicated by findings of associations with related retrospective assessments. Besides, we noted differences in EMA data between clinical groups and comparison groups, and the ability of EMA data to predict outcome or functioning. Nonetheless, given findings in, for example, youths with HFASD [22, 23], it seems important to adapt EMA to the abilities and experiences of the specific target group in order to achieve compliance and valid assessments. Moreover, the information provided by mobile applications versus phone calls may be further evaluated, as quick probes or follow-up questions can be added in phone calls when an unclear answer is provided [28]. Despite the idea that youths may be skilled in using mobile applications, phone calls that allow for answering verbally may be suitable for youth as well, also because literacy may vary in children and adolescents. However, by using images, adapted response scales, clear instructions, and training these concerns may be overcome (for a further discussion of methodological considerations, also see Heron et al. [16]).

In relation to compliance and validity, we also express some caution regarding the possibility of reactivity to the EMA protocol and EMA being perceived as burdensome (e.g., based on concerns raised by youths with psychosis [31]). Reactivity when using EMA to examine psychopathology refers to participants’ increased awareness of symptoms. This could be due to the frequent assessments, which could in turn result in a change in symptoms or behavior. Surprisingly, although some authors considered the possible influence of potentially predictable time-based EMA on behavior (e.g., [42]), we hardly found any EMA studies reporting on reactivity. The impact of participating in an EMA protocol has been investigated in adults with psychopathology, and a reduction in the severity of symptoms of posttraumatic stress over the EMA monitoring period was found, which was not found over an unmonitored control period [69]. Reactivity should be taken into account when interpreting findings, and should be reported on more often. In terms of burdensomeness, EMA can be considered more demanding of parents and their children than more conventional methods. As a result, the willingness or ability to complete assessments may decrease. When designing intensive or lengthy EMA protocols, the possible burden placed on parents and their children should be weighed against the benefit of richer data.

In line with previous research on cross-informant correspondence in clinical assessment (e.g., low-to-moderate correspondence [70]), parental EMA data of their child’s moods and behavior were found to differ from the child’s own EMA data. While parental EMA data may in some cases be more informative, both may be considered of value. The child’s age may also play a role, as adolescents generally have more self-insight than primary school-age children. For example, based on findings in children with ADHD, it could be suggested that parental momentary reports on their children with ADHD may be more reliable and valid than child momentary self-reports [26, 32]. Moreover, both in children with ADHD and in children with separation anxiety, parent reports were better predictors of the children’s emotional and behavioral difficulties and specific diagnosis than child reports [25, 29, 30]. On the other hand, among adolescent girls with ADHD, in general there was similarity between their self-reports of negative emotions and those of their mothers [27]. Ultimately, the decision to involve both parents and children in an EMA study on youth symptoms may best be based on the specific research questions of a study along with considering children’s developmental stages.

### Potential of EMA in Studying the Phenomenology of Youth Psychopathology

The results of this review showed several ways in which EMA studies may provide insights the phenomenology of youth psychopathology. First, we demonstrated how EMA can be used to gather data on the many daily environmental and interpersonal factors that cannot easily be imitated in a laboratory but may be important variables of interest (e.g., day-to-day social context, such as interactions with peers, and the relationship with positive affect [54], co-rumination during specific social interactions [46], and the actual occurrence and impact of fearful situations [50]). It should be noted that most studies conducted EMA outside of school hours, and relevant information could be missed. Studies should therefore consider the possibilities and added value of conducting EMA during school hours, even though doing this may be challenging or undesirable. However, the reviewed applications do confirm that by assessing youth in their natural environments, EMA may provide valuable and ecologically valid information about specific events, social and affective contexts, and the relations between them in the daily lives of youth. This information may add to the understanding of mechanisms that contribute to the development of psychopathology and may provide potential targets for prevention and treatment.

Second, results showed how EMA could minimize retrospective recall and the subsequent risk of recall biases by asking questions near real-time (e.g., in adolescents with HFASD [22]). More generally, findings suggest that EMA data may provide different and possibly less biased information than less frequent retrospective reports, and may offer researchers and clinicians an additional methodology for increasing the accuracy of the assessment and monitoring of stressors and other relevant measures in youth with developmental disorders or other mental disorders. It should be emphasized that retrospective reports are, of course, also relevant for specific research questions. Compared to ‘in the moment’ EMA reports, they may integrate multiple experiences or an evaluation of one’s overall experience.

Finally, results showed how studies used EMA to capture day-to-day or even within-day fluctuations in mood, behavior, and context, and temporal associations between these variables which may not be captured by more traditional methods (e.g., in ADHD [25, 32], and in AN [59]). Relatedly, we noted that EMA was also combined with physiological measures, such as actigraph-measured sleep to examine the bidirectional association between daily affect and sleep [19], and cortisol testing to examine cortisol diurnal activity [21] in youths with affective disorders. EMA thus appears suitable for providing information on these fluctuations and within-person variability of affect and related variables or contexts in youth with psychopathology, and allows for studying temporal associations. Thanks to the richness of within-person data, EMA could add to more conventional methods by providing insights into and predictions on individual patterns of, for example, affect (e.g., [28]).

The latter benefit was also demonstrated in studies that used EMA to study parent–child interactions by independently assessing mother and child over time (e.g., their anger levels [40]). By studying the temporal relations between the moods and behaviors of parents and their children with EMA, both favorable and unfavorable patterns in dyads or families with a child with psychopathology could be identified. This type of information may eventually assist in tailoring interventions designed to improve the child’s functioning specific to the patterns in a dyad or family.

### Potential of EMA in Evaluating Treatment Outcomes

Our review showed how EMA may allow for the close monitoring of the processes of change during treatment. Thereby, EMA could add to the understanding of the changes and factors that contribute to treatment outcome. Traditionally, treatment studies rely on pre- and post-treatment questionnaires. However, in this way, important information about the course of treatment may be missed. Several studies have used EMA to monitor and predict treatment response (e.g., [20, 64]). Eventually, individual patterns visible in the EMA data may assist in identifying which youth will likely benefit from which treatment and whether their treatment should be changed. As a final note, in addition to monitoring and predicting treatment effects, EMA may even add to treatment in itself. Ecological Momentary Interventions (EMI) [71] could provide real-time support, for example, by cuing youths to practice CBT skills in anxious situations [72] or sending out positive thought assignments when someone indicates feeling depressed. It has already been suggested that adults with depressive disorder receiving pharmacological treatment benefit from additional feedback derived from EMA, providing insight in personalized patterns and contexts of affect [73].

### Strengths and Limitations of This Systematic Review

A strength of our review is that, to the best of our knowledge, it is the first to provide a systematic review of the use of EMA in the broad range of youth with internalizing and externalizing disorders. In addition, it has shown how EMA methods can be used to provide feasible and valid assessments of the daily presentation and course of youth psychopathology, interactions of symptoms with contextual factors, including parent–child interactions, and the course and outcome of treatment.

A limitation of this review is the use of strict study selection criteria. Some interesting EMA studies, for example, on non-suicidal self-injury, were excluded because they did not fulfill the criterion of studying a clinical sample with a DSM-IV or DSM-5 diagnosis. Also, as a consequence of excluding non-clinical samples, we could not review the potential of EMA in the context of prevention, even though frequent monitoring of mood or behavior may be very useful for detecting youth at risk for mental health problems. Another potential limitation of the current review was that it included a narrative section on potentials of applying EMA that were shown in the studies, which has possible disadvantages as it involves a less defined synthesis of findings. Yet, it did allow us to synthesize the potential of the variety of EMA applications in more detail within a structure of three topics.

### Future Directions

Regarding future directions, we encourage other researchers to make use of the adaptability of EMA and the richness of EMA data and to further apply it in studying youth psychopathology covered, and also psychopathology not covered in this review. For example, despite being mentioned in the search terms, no studies were found on substance use disorders or posttraumatic stress disorder in youths; EMA could also be applied in studying these disorders in youth (e.g., by gathering real-life contextual data on use or symptoms), as has been done in adult populations (e.g., [74, 75]). EMA may provide more opportunities for researchers and clinicians than those already described in this review. First, some studies used EMA to examine interesting variables in the daily lives of youth but did not relate the variables to social and affective contexts. For instance, Primack et al. [45] adapted EMA to examine the media use of youths with MDD, but media use was not linked to other variables such as negative and positive affect before, during, and after use. Second, interesting applications that were not found in the included literature consider the use of event-contingent EMA to analyze externalizing problems or eating behaviors. In non-clinical samples, event-contingent designs have already been used in, for example, children with binge eating symptomatology [76]. Third, while temporal relations between maternal and child behavior and moods were examined using EMA in youth with ADHD, this was not yet found in youth with internalizing problems. Fourth, concerning data analysis, some studies computed the average of the within-person fluctuations of the assessed variables. However, EMA data allows for more elaborate analyses that consider these within-person fluctuations to reveal interesting information about youth psychopathology. We encourage all to profit from the wealth of data resulting from EMA, and to conduct more elaborate analyses of existing data when this has not yet been done.

## Summary

We reviewed 50 studies that used EMA to study youth with ADHD, ASD, mood disorders, anxiety disorders, eating disorders, or psychosis. In addition to providing a systematic review of the characteristics of current EMA applications in all youth psychopathology, we synthesized their potential with regards to (a) feasibility and validity, (b) studying the phenomenology of youth psychopathology, and (c) evaluating treatment outcomes. A variety of EMA techniques was applied in the included clinical groups with generally adequate compliance (ranging between 57 and 99%). Findings supported the feasibility and validity of EMA in studying youth psychopathology. The potential of EMA in studying phenomenology was demonstrated by the use of assessments to examine symptom fluctuations and the specific contexts and events in which they occur, and by indications of minimized recall bias. Moreover, by using both parent and child reports, associations and temporal links between parent and child moods and behavior could be examined. Similarly, it was shown how EMA was used for closely monitoring and predicting treatment response. Gaps in applications included the limited use of event-based EMA and parent-report EMA across disorders, as well as the use of EMA in other disorders than those in the reviewed studies. We suggested adapting the EMA protocol to the specific target group and taking into account possible concerns such as burden and reactivity. Eventually, for youth with psychopathology, EMA may assist in informing treatment decisions and identifying specific patterns, interactions, and contexts. With this review, we hope to have informed and inspired researchers and clinicians how to use and adapt EMA in clinical child and adolescent psychology and psychiatry.

## Supplementary Information

Below is the link to the electronic supplementary material.

Supplementary file1 (DOCX 26 kb)
